# Angle-Sensitive Detector Based on Silicon-On-Insulator Photodiode Stacked with Surface Plasmon Antenna

**DOI:** 10.3390/s20195543

**Published:** 2020-09-28

**Authors:** Anitharaj Nagarajan, Shusuke Hara, Hiroaki Satoh, Aruna Priya Panchanathan, Hiroshi Inokawa

**Affiliations:** 1 Graduate School of Science and Technology, Shizuoka University, Hamamatsu 432-8011, Japan; anithr.91@gmail.com; 2 Department of Electronics and Communication Engineering, SRM Institute of Science and Technology, Chennai 603 203, India; arunaprp@srmist.edu.in; 3 Graduate School of Integrated Science and Technology, Shizuoka University, Hamamatsu 432-8561, Japan; hara.shusuke.14@shizuoka.ac.jp (S.H.); satoh.hiroaki@shizuoka.ac.jp (H.S.); 4 Research Institute of Electronics, Shizuoka University, Hamamatsu 432-8011, Japan

**Keywords:** angle-sensitive pixel, phase matching condition, SOI PD, SP antenna, lensless imaging

## Abstract

We present a pixel-level angle sensitive detector composed of silicon-on-insulator (SOI) photodiode (PD) stacked with a gold surface plasmon (SP) antenna to affect the direction of the incoming light. The surface plasmons are excited in the grating-type SP antenna and enhance the diffraction efficiency of the grating. The diffracted light is coupled strongly with the propagation light in the SOI waveguide when the phase matching condition is satisfied. The phase matching takes place at a specific angle of light incidence, and the discrimination of the light based on the incident angle is achieved. As spatial patterns in the polar coordinate of the elevation-azimuth angles (*θ*, *ϕ*) of the incident light, we present the phase matching condition theoretically, the absorption efficiency in the SOI by simulation, and also the quantum efficiency of the SOI PD experimentally for different SP antennas of one-dimensional (1D) line-and-space (L/S) and two-dimensional (2D) hole array gratings under various polarization angles. 1D grating offers a polarization sensitive angle detection and 2D grating exhibits angle detection in two orthogonal directions, enabling a polarization independent angle sensitivity. A good agreement among the theory, simulation, and experiment are attained. The proposed device features relatively high quantum efficiency as an angle-sensitive pixel (ASP) and gives wider opportunities in applications such as three-dimensional (3D) imaging, depth-of-field extension, and lensless imaging.

## 1. Introduction

The current trend in the research field of advanced image sensors is to focus on the development of pixel-level detectors for computational imaging [1,2,3,4]. A traditional camera forms an image by recording only a two-dimensional (2D) intensity map of a scene. A light field camera or plenoptic camera records light field information, which provides a more complete description of a scene than a traditional camera. The light field is the collection of light rays flowing in every direction through every point in space. The light field is defined by the mathematical function called plenoptic function denoted by five-dimensional (5D) intensity data I(*x*, *y*, *z*, *θ*, *ϕ*) [5]. The 5D plenoptic function is the combination of the three spatial coordinates (*x*, *y*, *z*) and the two angle information (*θ*, *ϕ*) which represent elevation and azimuth angles, respectively, of the light ray. For simplicity, this function is reduced to 4D data I(*x*, *y*, *θ*, *ϕ*), by omitting *z*, as the intensity along a ray is a constant [6,7]. Several techniques have been developed to capture the local angular distribution from the light field for many interesting applications. For example, capturing the angle information allows computationally refocusing of the image during post-processing [8]. Furthermore, it also allows 3D imaging, lensless imaging, and so on [9,10,11,12,13,14,15]. Recently, a novel CMOS (complementary metal-oxide-semiconductor) image sensor has been developed to detect the angular information as a representative angular sensitive pixel (ASP). This sensor utilizes the Talbot effect between two diffraction gratings stacked over a conventional photodiode and it became possible to measure one angular information *I*(*θ*) of the incident light [16]. However, in order to complete a plenoptic function, it is indispensable to be able to sense two angle information, which includes elevation angle *θ* and azimuth angle *ϕ*. It was reported that this Talbot pixel has a problem of reduction in quantum efficiency (QE) of 88% compared to the bare photodiode and was improved to a 55% reduction rate after the post-fabrication process of ASP; however, it exhibits an angular resolution of ~8°. Moreover, the output response of Talbot pixel is sinusoidal and fades as the angle increases. It requires four pixels to decode single angle information. Hence, the development of CMOS compatible, easily fabricated, high QE possessing features with a less angular resolution and a capability of using a smaller number of pixels to decode single angle information is highly anticipated.

In this article, the performance of incident angle dependence of light sensitivity in our proposed silicon-on-insulator photodiode (SOI PD) with surface plasmon (SP) antenna [17,18,19,20] is investigated for one candidate of advanced ASPs. In addition, the incident angle dependence for different incident polarization is also clarified. Our proposed SOI PD with SP antenna basically utilizes the strong coupling between the diffracted light from the grating structure of the SP antenna and the waveguiding modes in the SOI layer. We have investigated the elevation-azimuth angle dependence of the light sensitivity in SOI PD with a SP antenna, but the polarization angle directly depends on the azimuth angle because of our previous measurement configuration with one axis goniometer stage [21,22]. Since such a situation is unnatural, a two-axis goniometer stage is introduced instead of a one axis goniometer stage to evaluate a full elevation-azimuth angle dependence for a specific polarization. In this case, the polarization angle is independent of the azimuth angle, and thus the investigation in this article is close to the actual situation for light detection.

We investigate the directivity of light sensitivity in SOI PD with two types of SP antenna composed of one-dimensional (1D) line-and-space (L/S) grating and 2D hole array grating by using theoretical prediction, electromagnetic simulation, and experimental demonstration. The structure of 1D grating is formed by the periodic arrangement of the Au line and space in one direction. The 2D grating is formed by the superimposition of the 1D grating stacked orthogonally to each other, thereby resulting in a periodic arrangement of holes in both of the directions. A theoretical prediction includes the formalization for representing the peak position for an elevation-azimuth angular distribution of incident light. The physical concept behind this theory is that a higher light sensitivity is achieved at a specific incident angle when the phase matching condition between the diffracted light from the antenna and the propagation light wave in the SOI waveguide is satisfied. The antenna primarily works as a grating coupler and exhibits the angle sensitive characteristics for orthogonal polarized light for the 1D LS grating. In order to realize angle sensitive characteristics with polarization independent behavior, 2D hole array grating is employed, which has periodicity in both of the directions. An electromagnetic simulation based on the finite-difference time-domain (FDTD) method can design and estimate the performances of SOI PD with SP antenna for not only peak positions of the incident angle but also light sensitivity. The monochromatic incident light is tilted and rotated along with the elevation and azimuth angles, *θ* and *ϕ*, respectively for calculating the light absorption efficiency of the device. The SP antenna over the light sensitive area of the SOI PD is fabricated by using an electron beam lithography technique. The experimental demonstration of the fabricated devices is done by measuring the directivity of quantum efficiency for different polarizations.

## 2. Device Structure and Fabrication Process

**Figure 1:**
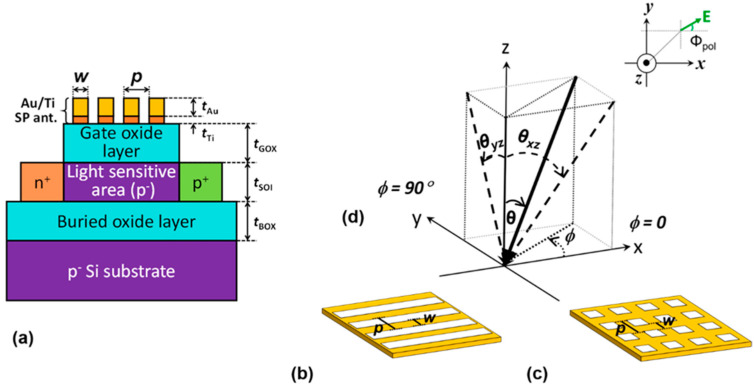
(**a**) Cross-sectional view of the silicon-on-insulator (SOI) photodiode (PD) with a surface plasmon (SP) antenna; (**b**) 1D line and space grating; (**c**) 2D hole array grating; (**d**) definitions of azimuth (*ϕ*), elevation (*θ)*, and polarization (*ϕ*_pol_) angles of the incident light reproduced from [22]. The projected elevation angles *θ_xz_ and* *θ_yz_* to *x*-*z* and *y*-*z* planes, respectively, are also clarified for theoretical discussions.

The device structure of the SOI PD with SP antenna is shown in Figure 1a. The lateral p-n junction SOI PD is formed by using commercial SOI wafer. In this work, the typical SOI thickness of *t*_SOI_ = 100 nm was used, since an SOI thickness of below 200 nm is widely used in SOI integrated circuits. The silicon dioxide (SiO_2_) layer on SOI PD, which works as a gate oxide, has the thickness of *t*_GOX_ = 100 nm. The primary role of the SP antenna is to act as a grating coupler between the incoming light and the lateral propagating light in the SOI layer. The *t*_GOX_ was optimized to achieve the maximum QE for an incident wavelength of 700 nm [17]. The QE is the ratio of the number of photogenerated carrier to the one of incident photons to the device. Without SP antenna, the SOI PD shows low QE due to the thin *t*_SOI_ for light absorption, but the QE can be enhanced by up to 25% by introducing the SP antenna with 1D L/S gold (Au) grating, as we previously reported [18]. This time, we introduced two types of SP antennas with L/S and hole array gratings, and clarified the difference between their characteristics. The L/S grating has a 1D periodic arrangement with a period *p* and a line width *w*, and the hole array grating has a 2D periodic arrangement of square holes in a square lattice with a period *p* and a line width *w*, as shown in Figure 1b,c, respectively. Au was chosen as the material for a SP antenna because Au has higher durability towards the surface oxidation than that of silver or aluminum. In order to obtain the sufficient adhesion strength between Au and SiO_2_, thin titanium (Ti) layer was inserted. The thicknesses of the Au and Ti used were *t*_Au_ = 50 nm and *t*_Ti_ = 5 nm, respectively. The SP antenna acts not only as a grating coupler between incident and laterally propagating waves in the SOI, but also as a gate electrode. When the device is irradiated, the diffracted light from the SP antenna excites the SOI waveguide mode. A large QE can be obtained only if the phase matching condition is satisfied, as will be discussed later. It is also important to apply appropriate bias voltages to the gate and the substrate so that the photocurrent can be maximized by expanding the depletion region to cover the entire light sensitive (p^−^) area [18]. The angle dependence in light sensitivity was investigated in this work. The definitions of elevation angle *θ*, and azimuth angle *ϕ* are shown in Figure 1d. In addition, the polarization angle *ϕ*_pol_ with respect to the grating direction is defined in this figure. The incident light is called TE-polarized and TM-polarized when the electric field component coincides with the x-direction (*ϕ*_pol_ = 0) and y-direction (*ϕ*_pol_ = 90°), respectively. The projected elevation angles on *x*-*z* and *y*-*z* planes, which are *θ_xz_* and *θ_yz_*, respectively, are the variables used in the explanation of the physical origin of the peak angle in Section 3, and are expressed as follows:

(1) $$\theta_{xz}={\;\tan}^{-1}\left(\tan\theta \cos\phi \right),$$

(2) $$\theta_{yz}={\;\tan}^{-1}\left(\tan\theta \sin\phi \right).$$

In this paper, the case is discussed for the first time where the polarization angle *ϕ*_pol_ is independent of *ϕ*, whereas *ϕ*_pol_ was rotated together with *ϕ* in our previous work [21,22].

**Figure 2:**
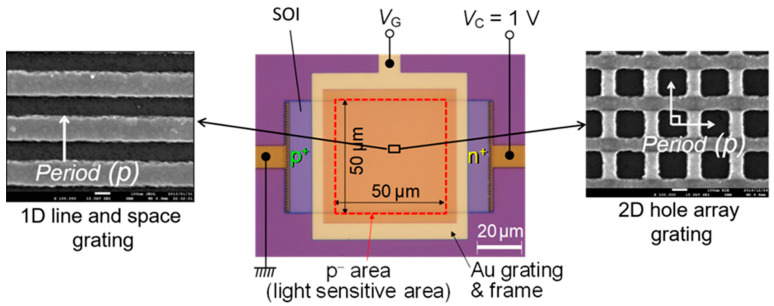
Top view (optical and scanning-electron micrographs) of the fabricated device with the 1D line and space grating and a 2D hole array grating reproduced from [22].

This SOI PD was fabricated by using the following steps. The first step was to adjust the thickness of the p-type SOI layer through thermal oxidation and removal of the oxide layer. The second step was to pattern the SOI layer by photolithography for the isolation of PDs. The third step was to implant the BF_2_^+^ and the As^+^ ions to form anode and cathode regions, respectively. The fourth step was to form a gate oxide on the SOI layer by using oxidation and chemical vapor deposition. The final step was to pattern the Au SP antenna by using electron-beam lithography and a lift-off process. The top view of the fabricated device with Au grating stacked over the light sensitive p^-^ area of 50 × 50 μm^2^ is shown in Figure 2. Since the SP antenna is surrounded by the frame, an electrical contact to the gate electrode is established. This fabricated device is compatible with the CMOS integrated circuit technology except for the usage of Au, and offers good manufacturability as a monolithic device with multiple PDs in a single chip.

## 3. Principle of Angle Detection

**Figure 3:**
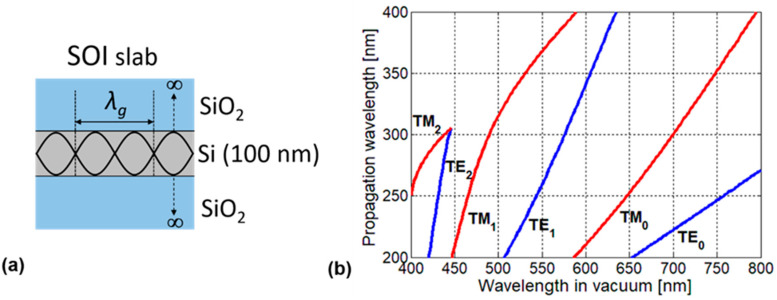
(**a**) The three-layered symmetrical slab waveguide consists of a 100-nm-thick Si core and SiO_2_ claddings; (**b**) Propagation wavelengths of the waveguide modes in the SOI layer calculated by the dispersion relation vs. the free space wavelength.

The basic principle of peak angle detection provided by the device is explained in this section. The SOI photodiode functions as a waveguide, as its structure is similar to a symmetrical slab waveguide where the Si layer acts as the core with high refractive index and the gate oxide and the BOX layer act as claddings with a low refractive index (SiO_2_). In Figure 3a, a representation of the waveguide modes in the SOI slab is shown. The main contribution of the SP antenna in the enhancement of light absorption is caused by the strong coupling between the enhanced diffracted light and the propagation mode in the SOI layer.

The angle-selective enhancement of the device occurs when the phase matching condition between the diffracted mode of the antenna and the propagation mode of the SOI waveguide is satisfied. Through the concept of phase matching condition and the relation between the grating period of the SP antenna, the waveguide mode of SOI PD, the wavelength, and the incident angle (*ϕ*, *θ*) of light could be formalized and used to predict the directivity of the SOI PD with the SP antenna towards the incoming light.

To analyze the basic principle in detail, we first considered the dispersion relation for the symmetrical Si-core slab waveguide. The propagation wavelength of the waveguide mode can be predicted by using the dispersion relation based on the following transcendental equations [23]:

For TE modes,

(3) $$\tan\left(\frac{ht_{core}}{2}-m\frac{\pi }{2}\right)=\frac{\sqrt{V^{2}-h^{2}t_{core}^{2}}}{ht_{core}}\;(m\;=\;0,\;1,\;2,\;\ldots ,)$$

For TM modes,

(4) $$\tan\left(\frac{ht_{core}}{2}-m\frac{\pi }{2}\right)=\frac{n_{core}^{2}}{n_{clad}^{2}}\frac{\sqrt{V^{2}-h^{2}t_{core}^{2}}}{ht_{core}}\;(m\;=\;0,\;1,\;2,\;\ldots ,)$$

where,

$$h=\sqrt{{\left(\frac{2\pi n_{core}}{\lambda }\right)}^{2}-{\left(\frac{2\pi }{\lambda_{g}}\right)}^{2}},\;\;\;V=\frac{2\pi }{\lambda }t_{core}\sqrt{n_{core}^{2}-n_{clad}^{2}}$$

*t*_core_ is Si core thickness (100 nm in our design), while *n*_core_ and *n*_clad_ are the refractive indices for Si core and SiO_2_ claddings, respectively. Especially for *n*_core_, the wavelength dependence is considered. λ and λ_g_ are the free space wavelength and the propagation wavelength, respectively. Figure 3b shows the calculated propagation wavelength of the waveguide in the visible light wavelength range.

**Figure 4:**
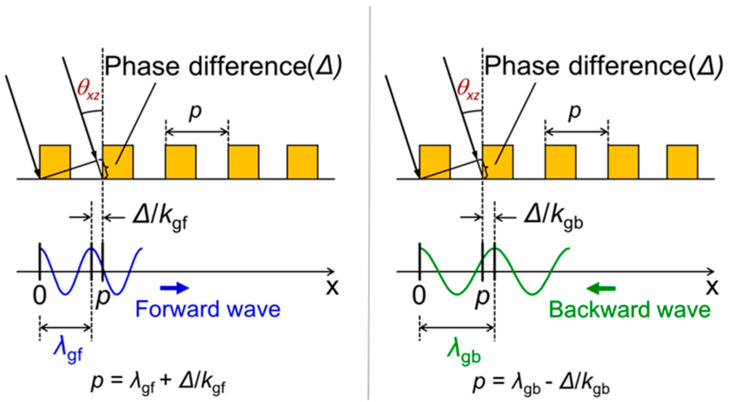
Phase matching conditions between the diffracted wave from antenna surface and the waveguide mode in the SOI layer for forward and backward waves reproduced from [22]. *k_gf_ = 2π/λ_gf_* and *k_gb =_ 2π/λ_gb_* are the wavenumbers for forward and backward waves, respectively. In the case of *y*-direction, *θ_yz_* is considered instead of *θ_xz_*.

We then considered a monochromatic incident light where the wavelength of *λ* is tilted in the *x-z* plane with the angle of *θ_xz_* and the peak angle could be obtained from the previously noted dispersion relation and the following phase matching condition [20]. Figure 4 shows the schematics of the phase matching condition between the diffracted light from SP antenna and the waveguiding mode in the SOI layer when the incident angle is elevated. Due to the periodic arrangement of gold line and space, phase difference Δ occurs between the rays entering the adjacent gold lines in the antenna, when the incident angle is tilted, and Δ is given by

(5) $$∆=p\left(2\pi /\lambda \right)\sin\theta_{xz}.$$

This phase difference is responsible for propagation of two different waves in the SOI waveguide: forward and backward waves. The forward and backward waves have shorter and longer wavelengths, respectively, with respect to the grating period of the antenna. At a specific angle, the SP antenna strongly couples with the diffracted light from the antenna surface to the propagating wave in the SOI layer when the phase matching condition is satisfied. This phase matching condition occurs at different angles correspondingly with the grating period for forward and backward waves. Based on this physical concept, the mathematical equations were modeled for predicting the peak angle in *x-z* plane is (*θ_xz_*) as follows:

For forward waves,

(6) $$\theta_{xz}=\sin^{-1}\lambda \left[\left(1/\lambda_{gf}\right)-\left(1/p\right)\right]$$

and for backward waves,

(7) $$\theta_{xz}=\sin^{-1}\lambda \left[\left(1/p\right)-\left(1/\lambda_{gb}\right)\right]$$

where *λ*_gf_ and *λ*_gb_ are the propagation wavelengths in forward and backward directions. These theoretical peak angles can be designed using the following procedure. At first, the propagation wavelength *λ*_g_ for the free space incident wavelength *λ* is obtained from the dispersion relation, as shown in Figure 3b. Substituting the calculated value *λ*_g_ for *λ*_gf_ or *λ*_gb_ in Equations (4) or (5), the peak angle *θ_xz_* of forward or backward waves can be obtained for each grating period at a fixed incident wavelength.

When the phase matching condition between the waves in the antenna and the SOI layer is considered in the *y*-direction, the same manner can be adopted and *θ_yz_* is considered instead of *θ_xz_*.

In the above equations, the elevation angle for a fixed azimuth angle could be predicted. i.e., when *ϕ* = 0 and 90°, *θ_xz_* and *θ_yz_* tilting occurs, respectively. For a 2D angular mapping, azimuth (*ϕ*) angle detection also needs to be precisely predicted in addition to the elevation (*θ*) angle. This can be calculated by considering Equations (1) and (2).

It is important to consider the design of both types of SP antenna in Figure 1b,c. The structure of 1D L/S grating has a periodic arrangement of line and space along the *x* direction, whereas along the *y* direction, a straight line is present. This phase matching condition effect appears only along the *x-z* plane, i.e., when the light direction is perpendicular to the grating orientation, whereas along the *y-z* plane, angle tilting has no effect on the optical phenomenon of detecting the angle, i.e., when the direction is parallel to the grating orientation. Thus, 1D grating would exhibit strong polarization sensitive angle detection.

In the case of 2D hole array grating, the periodic arrangement of holes is present in both the directions, *x* and *y* and due to its square latticed arrangement, the alignment of holes is also similar in both of the directions. Due to their symmetry, two phase matching conditions in *x*-*z* and *y*-*z* planes were imposed, as discussed in Section 4.2.

## 4. Results and Discussions

### 4.1. 1D L/S SP Antenna

**Figure 5:**
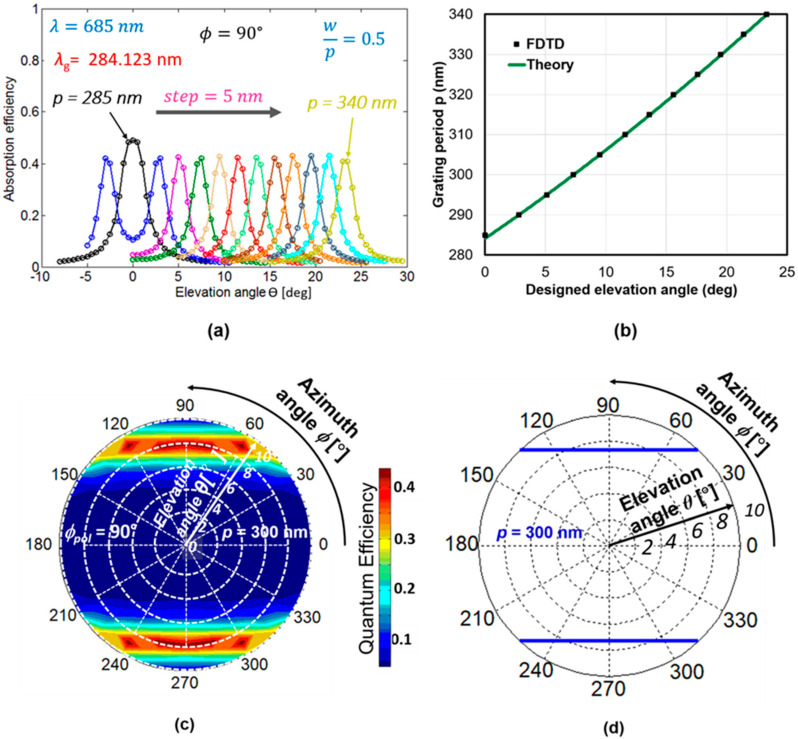
(**a**) Absorption efficiency as a function of the elevation angle in SOI PD with 1D L/S SP antenna (FDTD calculation) with various grating periods *p*; (**b**) Comparison of appropriate grating periods for designed peak elevation angle estimated by FDTD method and phase matching condition. (**c**) Calculated spatial pattern of absorption efficiency at *ϕ*_pol_ = 90° and (**d**) theoretical spatial pattern of peak incident angles based on the phase matching condition. Grating period is fixed at *p* = 300 nm.

We first show the light sensitivity in the SOI PD with 1D L/S SP antenna to the elevation angle (*θ*) tilting at a fixed azimuth angle (*ϕ*) and polarization angle (*ϕ*_pol_). To predict the performance of our devices quantitatively, the absorption efficiency in the SOI layer was calculated by using the FDTD method. In this simulation, we used the Lorentz–Drude oscillator model and the single-pole Lorentz model to introduce the dielectric constants depending on wavelength of metals (Au and Ti) and silicon, respectively. These parameters are the same as the ones in [18]. The relative permittivity of SiO_2_ for the layers of BOX and gate oxide were fixed at 2.13. In order to reduce the computation costs, the periodic boundary condition and the absorbing boundary condition were adopted. Especially for absorbing the boundary condition, we chose the perfectly matching layer (PML). FDTD simulations mainly evaluate the absorption efficiency based on the ratio of the power absorbed in the SOI layer to the one of incident light, because the absorption efficiency in the SOI layer corresponds to the external QE of PD when it is assumed that the internal QE of PD is unity. The absorption power in the SOI layer can be obtained by calculating the term of conduction loss in the Maxwell equation or by subtracting the power passing through at the lower interface of SOI from the upper interface [17]. Figure 5a shows the FDTD results of absorption efficiency in the SOI layer as a function of elevation angle *θ* for various grating periods *p* ranging from 285 to 340 nm with an interval of 5 nm. The incidence was a TM-polarized monochromatic light with the wavelength of 685 nm, and the incident direction was moved along the perpendicular one to the L/S orientation, i.e., at *ϕ* = *ϕ*_pol_ = 90°. An elevation angle dependence of absorption efficiency with a sharp peak was observed at each period, and the peak elevation angle clearly shifted due to the change of the grating period. The peak heights except for *p* = 285 nm were almost the same, and the peak height in the case of *p* = 285 nm was about 0.1 larger than the others. The propagation wavelength of the symmetrical waveguide with 100-nm-thick Si for the incident wavelength of 685 nm was 285.5 nm calculated from Equation (2). When the propagation wavelength and the grating period *p* of SP antenna is matched, strong coupling of the diffracted light with the SOI waveguide mode occurs for normal incidence. This characteristic has been already discussed in detail in our previous research [17,18,19,20]. In Figure 5b, the peak elevation angle for the different grating period *p* under the TM waveguide mode was predicted by the FDTD calculation and phase matching condition for the forward waves in order to check their correlation. Similar trends were obtained so that peak elevation angle linearly increases as the grating period *p* increases and the peak positions coincide exactly with each other. The output response of each pixel could be modeled by using a simple Gaussian distribution equation. Thus, it can be shown that *p* can tune the peak elevation angle. The peak angle could also be controlled by varying the thickness of the SOI layer. However, we fixed the *t*_SOI_ to facilitate monolithic integration of PD with different grating periods for achieving angle detection with different characteristics. Note that the phase matching condition can estimate a peak elevation angle only, but FDTD results can investigate more details, including absorption efficiency, peak width, and so on. Considering the actual incident angle detection, we had to discuss not only the elevation angle but also the azimuth angle. In addition, the light in nature consists of polarized rays. Thus, the elevation-azimuth angle dependence of QE for different types of polarization was investigated for a rich collection of light information for image processing applications. A distribution of absorption efficiency based on polar coordinate system was used as a spatial pattern. This spatial pattern is convenient to represent the complete spherical optical information emanating from a point in space. Figure 5c shows the FDTD results of the spatial pattern at *p* = 300 nm and *ϕ*_pol_ = 90°. Figure 5d shows the peak position of azimuth and elevation angles based on the phase matching condition. The spatial pattern based on the phase matching condition has good agreement with the one estimated by FDTD calculation, with a small angle deviation of ~0.5°.

**Figure 6:**
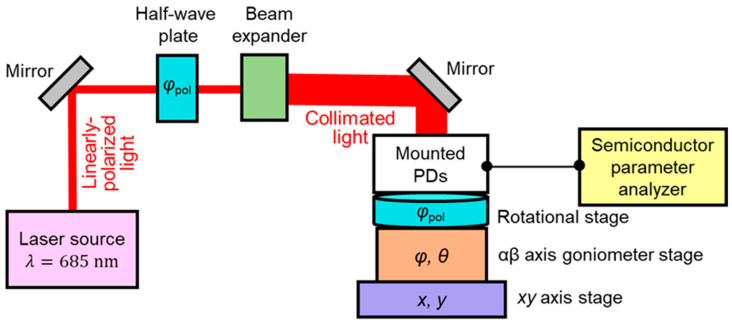
Measurement system for directivity of external QE in SOI PD with a SP antenna.

**Figure 7:**
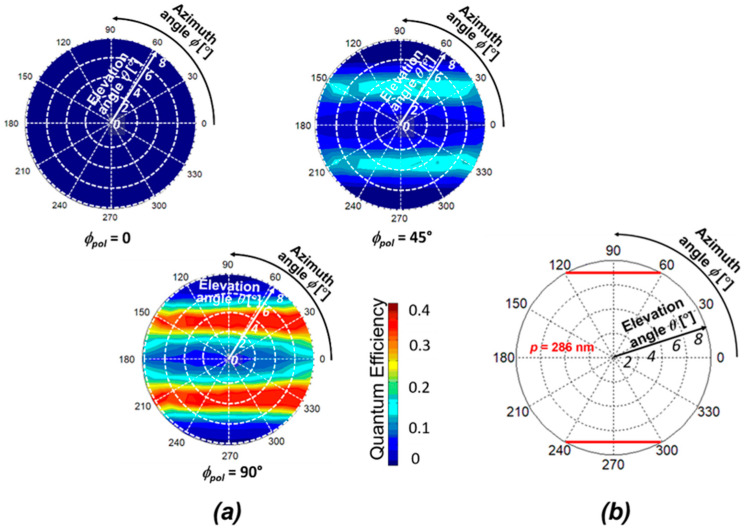
(**a**) Measured spatial patterns in SOI PD with a 1D L/S SP antenna for different polarizations (i) *ϕ*_pol_ = 0, 45° and 90°; (**b**) theoretical spatial pattern of peak incident angle based on phase matching condition to verify the measurements reproduced from [22]. The grating period was fixed at *p* = 286 nm.

The SOI PD with 1D L/S antenna is fabricated for measurements, where the thicknesses of SOI, gate oxide, BOX, Au, and Ti are *t*_SOI_ = 105 nm, *t*_ox_ = 100 nm, *t*_BOX_ = 400 nm, *t*_Au_ = 50 nm, and *t*_Ti_ = 5 nm, respectively. The grating period and width are *p* = 286 nm and *w* = 143 nm, respectively. The spatial pattern was measured for the elevation angles from 0 to 8° and the azimuth angles from 0 to 360° under three different polarizations: *ϕ*_pol_ = 0, 45°, and 90°. Figure 6 shows our measurement system. The light source is the solid-state laser, which emits a linearly-polarized light with a wavelength of 685 nm. The half-wave plate was used to calibrate a basic axis for the incident polarization angle *ϕ*_pol_ from the grating orientation of PDs, and *ϕ*_pol_ was adjusted by the rotation stage. The collimated light by beam expander was irradiated to the PDs. The azimuth angle *ϕ* and the elevation angle *θ* were adjusted by moving the αβ goniometer stage. The electrical characteristics of fabricated PD were measured by using a semiconductor parameter analyzer. Here, the photocurrent is evaluated when the reverse bias voltage of −1 V is applied between the anode (p^+^ region) and the cathode (n^+^ region) electrodes. Since our metallic SP antenna can be used for a gate electrode, the gate bias voltage was applied to control the carrier distribution. Combined with the substrate bias voltage, the depletion region can be expanded entirely in a p^−^ light sensitive region [18]. In this paper, the gate voltage at *V*_G_ = 7 V and the substrate voltage at *V*_SUB_ = −25 V are commonly used, including in the next subsection. The measured spatial patterns for different polarization angles (i) *ϕ*_pol_ = 0, (ii) 45° and (iii) 90° are shown in Figure 7a. The contour map with two-fold symmetry appeared in all the cases. When the incident light with the electric field perpendicular to the grating orientation (TM polarization) is illuminated, strong coupling occurs with the TM fundamental waveguide mode (TM_0_) in the SOI layer [21,22,23,24]. The QE reaches a maximum of 0.4 as predicted in the FDTD results when *ϕ*_pol_ = 90°, and reaches 0.2 (half value in the case of *ϕ*_pol_ = 90°) when *ϕ*_pol_ = 45°. Thus, the QE is higher in the case of perpendicular polarization and gradually decreases as the polarization decreases to zero. Such polarization dependence has been already described in our previous work, and the maximum QE for each polarization angle in a spatial pattern, denoted by *η*_max_ (*ϕ*_pol_), can be roughly estimated by *η*_max_ (*ϕ*_pol_) = *η*_max_ (*ϕ*_pol_ = 90°) cos^2^*ϕ*_pol_ [22]. Figure 7b shows the theoretical spatial pattern for the grating period *p* = 286 nm based on the phase matching condition. It is shown that the two-fold symmetry was also obtained that was similar to the experimental demonstration. For the case of *ϕ*_pol_ = 0, (TE polarization), the coupling occurs with the TE waveguide modes propagating in the SOI slab. However, the peaks corresponding to this polarization are invisible in the elevation angle range of these measurements.

### 4.2. 2D Hole Array SP Antenna

**Figure 8:**
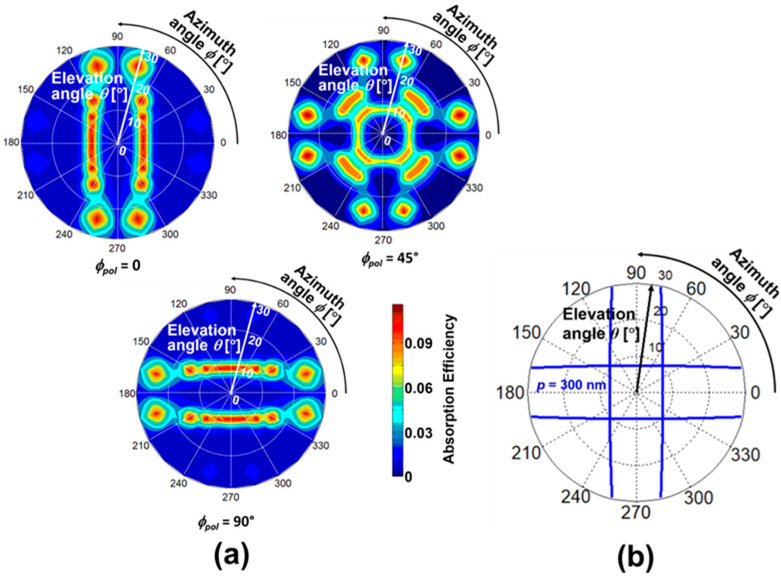
Numerical prediction of SOI PD with a 2D SP antenna. Polarization dependence of 2D angular detection in SOI PD with a 2D hole array grating with the period *p* = 300 nm. (**a**) FDTD spatial pattern showing 2D angular mapping for different polarizations, *ϕ*_pol_ = 0, 45°, and 90°; (**b**) theoretical spatial pattern for SOI PD for a 2D hole array with a grating period of *p* = 300 nm reproduced from [22].

Here, the type of SP antenna was changed to a 2D hole array, and its spatial pattern depending on incident polarization is discussed. As discussed in the previous subsection, the FDTD calculation was performed before obtaining measurements for the PD with a grating period of *p* = 300 nm and SOI thickness of *t_SOI_* = 100 nm. Figure 8a shows the spatial pattern for different polarization of *ϕ*_pol_ = 0, 45°, and 90°. In the cases of *ϕ*_pol_ = 0 and 90°, the FDTD spatial patterns have two-fold symmetry in the vertical and the horizontal directions, respectively. However, in the case of *ϕ*_pol_ = 45°, the spatial pattern with four-fold symmetry appears. This pattern may be caused by the superposition of the patterns of *ϕ*_pol_ = 0 and 90°. The peak elevation angle trend in *ϕ*_pol_ = 90° is the inverse of that of the *ϕ*_pol_ = 0 as the azimuth angle increases. Thus, peak angle sensitivity is highly symmetric with the parallel, and perpendicular calculation is also further expanded to predict the 2D angular distribution and plotted in polar coordinates with the spatial pattern representation, as shown in Figure 8b. The elevation-azimuth angular information mapping by the theory and numerical predictions were in good agreement, ensuring that the peak angle appears based on the optical concept and phase matching condition.

**Figure 9:**
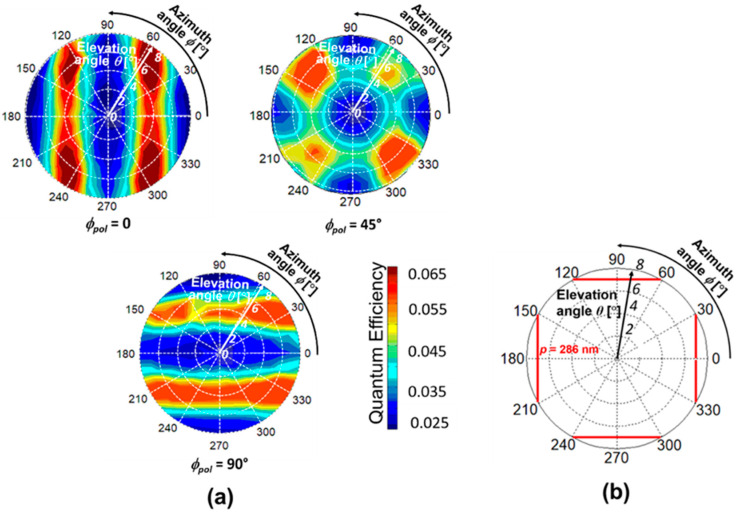
(**a**) Measured spatial patterns in SOI PD with a 2D hole array SP antenna for different polarizations of (i) *ϕ*_pol_ = 0, 45°, and 90°; (**b**) theoretical spatial pattern of a peak incident angle based on phase matching conditions to verify the measurements reproduced from [22]. The grating period is fixed at *p* = 286 nm.

**Figure 10:**
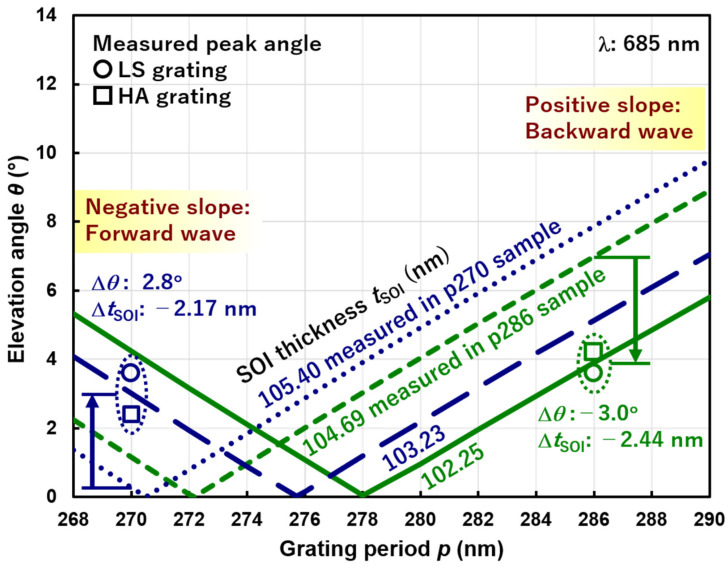
Comparison between measured peak elevation angles (circles and squares indicate LS and HA grating, respectively) and theoretical lines based on the phase matching condition. Samples with the grating periods *p* = 270 (blue) and 286 (green) nm have different measured SOI thicknesses *t*_SOI_ = 105.40 and 104.69 nm, (dotted lines) respectively. Theoretical lines for measured and estimated (solid lines) SOI thicknesses for *p* = 270 and 286 nm are shown. The arrows indicate the shift of measured angles from the estimated ones.

For the demonstration of polarization dependence in SOI PD with 2D hole array to elevation-azimuth angle detectivity, the external quantum efficiency was measured for the polarizations, *ϕ*_pol_ = 0, 45, and 90° and the corresponding spatial patterns are represented in Figure 9a. The polarization 0 and 90° shows a two-fold symmetry and follows the inverse trend of peak sensitivity with each other. Due to the high symmetric structure of the 2D SP antenna, strong coupling occurs for both the parallel and perpendicular polarizations. Also, the polarization 45° exhibits a superposition of the polarizations 0 and 90°. The experimental spatial pattern of SOI PD with 2D hole array SP antenna trend is similar to that observed in the numerical prediction. The theoretical spatial pattern for SOI PD with 2D SP antenna with *p* = 286 nm and *t_SOI_* = 105 nm for the TM mode is shown in Figure 9b. The peak angles of the theory and the experiment are close enough with a small variation, which is explained in Figure 10. The spatial patterns for both the devices clearly show the capability to detect both azimuth and elevation angles and polarization of the incoming light. Each device is capable of decoding an angular information with a high angular resolution of ~2°. The spatial pattern shows the full sphere of optical information impinging on a point in space and reveals the angle detection in both elevation and azimuth directions. LS grating owing to its 1D periodicity with high QE (45%) exhibits a strong polarization sensitive behavior and stands as a promising candidate for developing pixels for polarization vision applications. Hole array grating has the advantage of detecting angles with polarization independent behavior.

Figure 10 compares the measured peak elevation angles (indicated by symbols as shown in the legend) for two grating periods of *p* = 270 (blue) and 286 (green) nm, and grating types of 1D L/S (circle) and 2D hole array (square) with the theoretical lines based on the phase matching condition for measured (dotted lines) and estimated (solid lines) *t*_SOI_. The SOI thicknesses were measured by the spectroscopic reflectometer (Otsuka Electronics Co., Ltd., Tokyo, Japan, FE-3000) just before the fabrication of the SP antenna. The measured peak angles are 2.8° larger and 3.0° smaller than the theoretical ones for *p* = 270 and 286 nm, respectively. These deviations from the theory can be explained by the reduction of *t*_SOI_ by 2.17 and 2.44 nm, respectively. Note that the decrease in *t*_SOI_ results in the increase and the decrease of the peak elevation angles for forward and backward waves, respectively. In order to understand the discrepancy in *t*_SOI_, optical constants of Si and SiO_2_ in the wavelength range of 230–800 nm used in the *t*_SOI_ measurement need to be analyzed as a future task. These results indicate that the accurate control of *t*_SOI_ is necessary to reproduce the peak incident angles, which is not so difficult for advanced SOI CMOS technology. Also note that the material, i.e., optical constants, width, and thickness of the SP antenna do not affect the peak angles [19], which is beneficial for attaining reproducibility.

## 5. Conclusions

The effect of polarization on detectivity of light sensitivity in our proposed SOI PD with two types of SP antenna composed of 1D L/S grating and 2D hole array grating was investigated by using theoretical and simulated predictions, and experimental demonstration. While the conventional ASP focuses only on the local angular distribution of light, our proposed devices are capable of exhibiting elevation-azimuth angular detection. In addition, the spatial pattern representation was very unique for understanding the dependences of not only the elevation-azimuth angle but also polarization. The device with 1D grating shows angle detection with strong polarization selectivity. The 2D grating which was designed by simply changing the layout of the antenna by overlapping the 1D grating in two orthogonal directions, which exhibits angle detection with polarization insensitive behavior. The SP antenna played an effective role in affecting both the incident angle and polarization of light caused by the coupling between the diffracted light from the SP antenna and the waveguide mode in the SOI layer when the phase matching condition was satisfied. Several pixels with different grating periods could be integrated in a single chip for tuning multiple incident angles for sufficiently extracting the angle features of the image, and contributed to the development of an advanced ASP as a key component of plenoptic cameras that enable interesting applications such as three-dimensional (3D) imaging, depth-of-field extension, lensless imaging, and so on. 1D grating especially opens an opportunity for developing tiny pixels to enable applications based on polarization vision due to its polarization sensitive behavior.
